# Nano X Image Guidance in radiation therapy: feasibility study protocol for cone beam computed tomography imaging with gravity-induced motion

**DOI:** 10.1186/s40814-023-01340-z

**Published:** 2023-06-13

**Authors:** Emily Debrot, Paul Liu, Mark Gardner, Soo Min Heng, Chin Hwa Chan, Stephanie Corde, Simon Downes, Michael Jackson, Paul Keall

**Affiliations:** 1 Faculty of Medicine and Health, ACRF Image X Institute, The University of Sydney, Camperdown, NSW Australia; 2 Nelune Comprehensive Cancer Centre, Prince of Wales Hospital, Randwick, NSW Australia

**Keywords:** Image-guided radiotherapy, Image reconstruction, Cone beam CT

## Abstract

**Background:**

This paper describes the protocol for the Nano X Image Guidance (Nano X IG) trial, a single-institution, clinical imaging study. The Nano X is a prototype fixed-beam radiotherapy system developed to investigate the feasibility of a low-cost, compact radiotherapy system to increase global access to radiation therapy. This study aims to assess the feasibility of volumetric image guidance with cone beam computed tomography (CBCT) acquired during horizontal patient rotation on the Nano X radiotherapy system.

**Methods:**

In the Nano X IG study, we will determine whether radiotherapy image guidance can be performed with the Nano X radiotherapy system where the patient is horizontally rotated while scan projections are acquired. We will acquire both conventional CBCT scans and Nano X CBCT scans for 30 patients aged 18 and above and receiving radiotherapy for head/neck or upper abdomen cancers. For each patient, a panel of experts will assess the image quality of Nano X CBCT scans against conventional CBCT scans. Each patient will receive two Nano X CBCT scans to determine the image quality reproducibility, the extent and reproducibility of patient motion and assess patient tolerance.

**Discussion:**

Fixed-beam radiotherapy systems have the potential to help ease the current shortfall and increase global access to radiotherapy treatment. Advances in image guidance could facilitate fixed-beam radiotherapy using horizontal patient rotation. The efficacy of this radiotherapy approach is dependent on our ability to image and adapt to motion due to rotation and for patients to tolerate rotation during treatment.

**Trial registration:**

ClinicalTrials.gov, NCT04488224. Registered on 27 July 2020.

## Background

Radiotherapy plays a crucial role in the treatment of cancer patients, with just under half indicated to benefit from its inclusion in their treatment regime [1]. Cancer incidence is predicted to grow from 18.1 million cases reported in 2018 to 29.4 million by 2040. This increased incidence translates to greater demand for radiotherapy for which there is already a global shortfall, particularly in low- and middle-income countries [2]. Substantial investment is required to grow the global number of megavoltage radiotherapy units from around 14,000 to the estimated 21,800 required to meet demand by the year 2035 [3]. Furthermore, global geographic disparities in cancer outcomes exist, such as the correlation between radiotherapy machine availability and the gross income per capita as well as poorer cancer-reported outcomes in low- and middle-income countries. In order to meet growing demand and increase global access to radiotherapy, particularly for low- and middle-income countries and for rural areas in high-income countries, compact and low-cost radiotherapy solutions may be necessary.

**Fig. 1:**
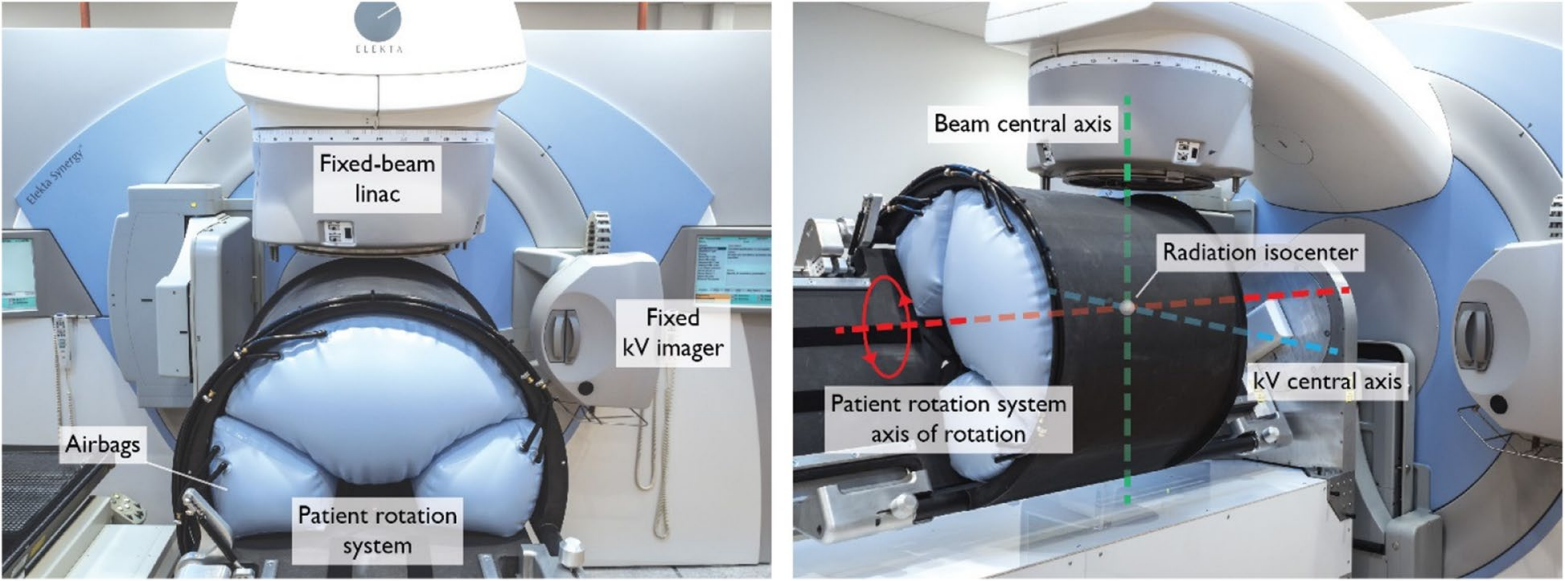
The components (left) and geometric configuration (right) of the prototype Nano X linear accelerator

Fixed beam radiotherapy systems, where the primary treatment beam is kept stationary and the target is moved and rotated, are extensively used in particle beam therapy to reduce the size and cost of the systems. The same concept could be applied to conventional X-ray linear accelerators to assist with meeting global radiotherapy demand. A small, economic ‘Nano X’ linear accelerator has been designed, prototyped and investigated based on this concept (Fig. 1) [4–8]. The Nano X has a novel design that relies on software to compensate for patient motion during imaging and treatment. Unlike conventional radiotherapy system where the heavy gantry is rotated, the Nano X design applies the inverse approach to conventional radiotherapy systems by using horizontal patient rotation and a fixed, vertical treatment beam. As the patient is expected to move during horizontal rotation, X-ray imaging is utilized to verify the patient position before and during treatment and adapt the radiation beam if necessary. This design provides simplification to the linac and a reduction in the bunker size and shielding requirements.

The Nano X radiotherapy system faces two primary challenges: (1) imaging and treatment delivery in the presence of anatomical deformation due to patient rotation and (2) patient acceptance and tolerance of rotation. This study aims to evaluate the feasibility of volumetric imaging for treatment guidance using cone beam CT (CBCT) on the Nano X fixed-beam radiotherapy system. In this case, the patient is rotated around the horizontal axis, and the kV imaging system is held stationary. The feasibility of the Nano X CBCT imaging will be evaluated by comparing the image quality of the Nano X CBCT reconstructions to conventional CBCT reconstructions. Furthermore, we aim to quantify the patient response to horizontal rotation.

## Methods

**Fig. 2:**
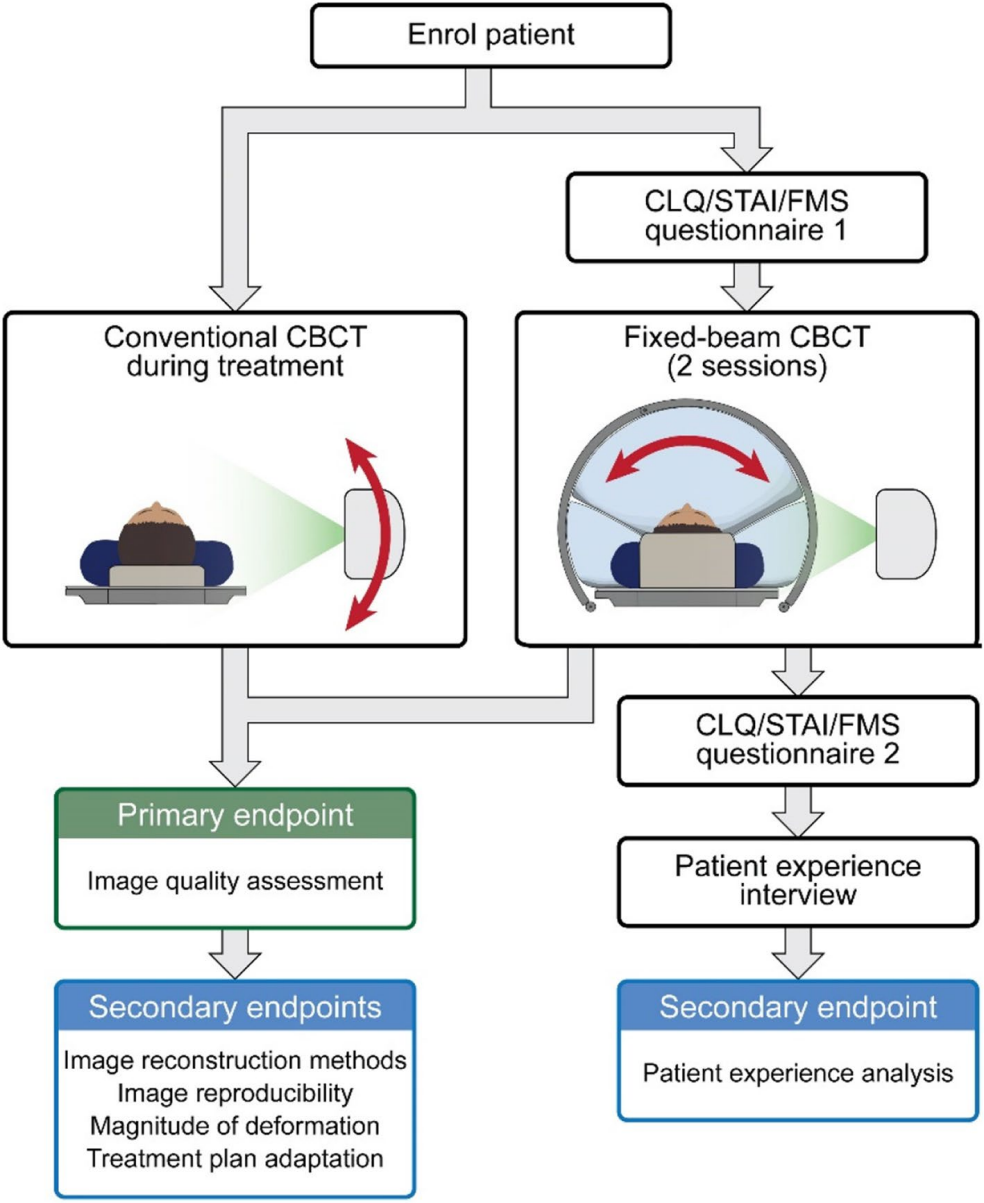
The study schema for the Nano X Image Guidance study. The CLQ, STAI and FMS questionnaires ask about claustrophobia, anxiety and motion sickness, respectively

Volumetric patient imaging with CBCT requires the X-ray projections to be acquired from a wide range of angles. This is achieved in conventional radiotherapy systems by rotating an X-ray source around the patient. The feasibility of CBCT imaging on the Nano X radiotherapy system, where the X-ray source is stationary and the patient is rotated horizontally, will be assessed in this single-institution, clinical imaging study. This study will recruit 30 radiotherapy patients at Nelune Comprehensive Cancer Centre (NCCC) at Prince of Wales Hospital (POWH). In addition to a conventional CBCT, patients will receive a CBCT scan while undergoing horizontal rotation on the prototype Nano X radiotherapy system. Patient images acquired with these two methods will be assessed for image quality by a group of qualified observers. The study schema is shown in Fig. 2.

The prototype Nano X consists of an Elekta Synergy linac at a fixed gantry angle of 0° and a bespoke patient rotation system (PRS). The Nano X prototype has been developed and extensively tested against 161 design, safety and usability specifications in accordance with the appropriate standards pertaining to the performance and safety of medical electrical equipment and medical linear accelerators [6]. The system was commissioned as reported by Liu et al. [8].

### Study design

This study is a single-arm blinded comparison study. The study involves up to three additional CBCT scans to be acquired for each patient, two of which will be performed on the Nano X radiotherapy system with the participant horizontally rotated and the kV imaging system held stationary (Nano X CBCT scan). Nano X CBCT scans will be used for study purposes only; patients will be treated on a conventional radiotherapy system. Study participants will be asked to attend an imaging session following two of their standard-of-care radiotherapy treatment fractions. During each of two study visits the participant will receive one Nano X CBCT scan.

- For participants who receive a conventional CBCT scan for image guidance during their standard-of-care radiation therapy treatment, this imaging data will be collected for study analysis and image quality comparator. These participants will receive a total of two CBCT scans (Nano X CBCT scans) in addition to standard treatment procedures over the course of the study.
- For participants who do not receive a conventional CBCT scan for image guidance during their standard-of-care treatment delivery (e.g. for breast cancer treatment where MV imaging is often employed), one additional conventional CBCT scan will be performed during their treatment fraction on the same day as one of the Nano X CBCT scans. These participants will receive a total of three CBCT scans in addition to standard treatment procedures (1 conventional and 2 Nano X CBCT scans).

The projections from the Nano X CBCT scan will be reconstructed offline following the participant visits.

The feasibility of using Nano X CBCT scans for volumetric image guidance will be assessed by comparing the image quality to a conventional CBCT scan of the same participant from their routine treatment (see the ‘Primary endpoint and assessment’ section).

The claustrophobia questionnaire will be administered only once before the first study session. Prior to each Nano X CBCT scan, patients will complete the anxiety and motion sickness questionnaires. Patients will then be immobilised on the Nano X PRS using safety strapping and computerised airbag restraints. The patient will undergo one continuous horizontal rotation that will take up to 3 min to complete. A Nano X CBCT scan will be acquired during the rotation. Following the scan, patients will again complete the anxiety and motion sickness questionnaires. Patients will undergo a semi-structured interview after their second session to assess their experience.

Data collected from the patients’ standard of care radiation therapy include patient demographics, treatment planning CT/4DCT, orthogonal planar kV images/CBCT/4DCBCT scans, treatment plans and structure set containing contours.

### Sample size

This study is a pilot feasibility study, and prior data is unavailable for sample size estimation. Therefore, a sample size of 30 was determined based on the feasibility of participant recruitment, the necessities of examining feasibility [9] and diminishing returns in precision for sample sizes exceeding this value [10].

### Key selection criteria

#### Inclusion

Cancer patients, aged 18 years or older, who are receiving radiation therapy and are able to be imaged using the Nano X PRS, with one of the following tumour sites: (a) in the head/neck region, (b) in the chest/abdomen region with fast CBCT used for standard of care image guidance and aged 60 years or older, (c) in the chest/abdomen region not receiving CBCT for standard of care image guidance and aged 70 years or older or (d) diagnosis of lung cancer with stage II-IV. ECOG Performance Status 0–2. The ability to communicate fluently in English to facilitate safe on- and off-boarding of the PRS and communication of comfort or distress to operators of the equipment throughout the session.

#### Exclusion

Patient positioning limitations of the Nano X PRS mean that tumour targets outside of the head/neck region and as far down as the abdomen cannot be imaged are not included. Patients outside the age restrictions stated above are excluded to due to legislated dose limits [11]. Patients with severe vertigo or with a recent diagnosis of benign paroxysmal positioning vertigo, weight > 137 kg (weight restriction of couch), pregnant women and patients from whom attaining informed consent would not be possible, including mental impairment, are also excluded.

### Primary endpoint and assessment

The primary objective of this pilot stage study is to determine whether CBCT volumetric image guidance is feasible for radiotherapy on a fixed-beam radiotherapy system using horizontal patient rotation. Feasibility will be assessed by comparing the image quality of reconstructed Nano X CBCT scans against conventional CBCT scans.

To measure the success of this objective, a combination of both objective and subjective image quality metrics will be used. For the subjective image quality metric, a group of experienced observers will perform image quality evaluations on a patient’s conventional CBCT scan and a Nano X CBCT scan side-by-side with 3 orthogonal views while blinded to the acquisition method. The image quality will be graded considering geometric accuracy, anatomical visualisation, sharpness of the image, soft tissue contrast, image noise and uniformity and overall clinical usability. Observers will be asked to indicate whether the image sets are of sufficient quality for image guidance, to select which CBCT scan set they deem to have superior image quality and whether it is of comparable, slightly superior or far superior image quality to the second CBCT scan. Observer responses will be assigned a score on a 5-point scale according to the study of Gardner et al. [12]. This metric is a subjective measurement of image quality; however, unlike quantitative image quality metrics, the observer scores provide a clinically relevant assessment of image quality and are robust to changes in patient anatomy between CBCT scans.

For the objective image quality metrics, a combination of standard metrics will be used, including signal-to-noise ratio (SNR), contrast-to-noise ratio (CNR) and edge-response width (ERW). These metrics evaluate both the spatial accuracy of the reconstructed CBCT images, as well as the reconstruction quality. The combination of both the objective and subjective image quality metrics provide a robust method for evaluating the image quality in a way that is robust and clinically relevant.

### Statistical analysis

Volumetric image guidance will be considered feasible for Nano X CBCT scans if the majority of reconstructed Nano X CBCT scans are either (1) deemed to have equivalent image quality to a corresponding conventional CBCT (image quality scores ≤ 3) or (2) the image quality is deemed sufficient for image-guided radiotherapy by a group of experienced observers. In this case, the majority is defined as a pass rate including uncertainty interval (one standard deviation) greater than 66% (e.g. 21 of the 30 participants Nano X CBCT scans pass).

### Secondary endpoints and assessments

Secondary objectives are as follows:

1. To quantify the patient experience of horizontal rotation on the Nano X PRS.
2. To refine motion-compensated image reconstruction methods to reduce motion artefacts and allow target and normal tissue visualisation at all treatment angles to a clinically acceptable standard
3. To determine the reproducibility and variability in Nano X CBCT compared to conventional CBCT
4. To quantify the magnitude of gravity-induced anatomical translation and deformation
5. To evaluate methods of treatment plan adaptation that compensate for gravity-induced target motion observed in Nano X CBCT scans

A mechanistic image reconstruction approach is necessary to account for gravity-induced motion during patient rotation resulting in both rigid and deformable anatomical movement. The aim of these reconstruction methods is to reduce the effect that motion has on the CBCT reconstruction, allowing for clear and geometrically accurate images that can assist with image guidance therapy. Studies conducted by Feain et al. [5], Shieh et al. [13], Liu et al. [14] and Gardner et al. [12] have demonstrated several image reconstruction methods that can correct for such gravity-induced motion. The Nano X CBCT projection data set will be used to evaluate the efficacy of similar image reconstruction methods in reducing motion blur artefacts and producing clinically useable volumetric images for treatment guidance. Quantitative image quality metrics such as the signal-to-noise ratio, contrast-to-noise ratio and edge response width will be used to compare volumes reconstructed with varying methods.

The reproducibility of Nano X CBCT acquisition will be assessed over the two sessions by comparing Nano X CBCT data sets at various reconstructed angles and will be benchmarked against conventional CBCT variability from daily image guidance during their treatment. The reproducibility and variability will also be evaluated using objective and quantitative image metrics including the signal-to-noise (SNR) ratio, contrast-to-noise ratio (CNR) and edge-response width (ERW). These metrics evaluate how well different anatomical features appear on the CBCT images and can hence be used to evaluate how closely the Nano X CBCT reconstructions match the conventional CBCT reconstructions. Hence, the reproducibility and variability will be evaluated using a combination of the objective and subjective image quality metrics.

The magnitude of gravity-induced patient motion will be measured by reconstructing the patient anatomy at eight rotation angles (every 45°). These volumes will be axially rotated back to the supine position and both rigidly and deformably registered to the 0° reconstructed volume. The rigid and deformable registrations will provide an estimate of the magnitude of the motion caused by patient rotation. The rigid and deformable components of patient motion will then be assessed similarly to Barber et al. [15] and Buckley et al. [16].

Treatment planning studies will be conducted by deforming the treatment planning CT and patient contours to the reconstructed anatomy from the Nano X CBCT at the given treatment angle. We have previously shown in a proof-of that improving the accuracy of CBCT images will improve the accuracy of radiation therapy treatment. The level of treatment plan adaptation required to compensate for observed patient and target motion will be evaluated and the dosimetric impacts of no adaptation versus iso-centre shift and full plan re-optimisation will be compared in a similar fashion to Buckley et al. [17].

Patients will complete psychometrically validated questionnaires assessing (1) their level of claustrophobia, (2) their level of anxiety and (3) their motion sickness related to horizontal rotation throughout the study using the Claustrophobia Questionnaire (CLQ) [18], short-form State/Trait Anxiety Inventory (STAI) [19] and Fast Motion Sickness (FMS) [20] questionnaires, respectively.

### Statistical analysis

The Wilcoxon signed rank test will be used to detect statistically significant differences in pre- and post-rotation scores of anxiety and motion sickness. The correlation between anxiety and motion sickness with claustrophobia will be assessed using Spearman’s rank correlation coefficient.

## Discussion

The Nano X Image Guidance study aims to assess the feasibility of performing volumetric image guidance with CBCT on a prototype fixed-beam radiotherapy system with horizontal patient rotation. While this type of system has the potential to provide a unique and affordable solution to improve global access to radiotherapy, demonstrating efficacy relies on evidence to support the three key assumptions. First, the target and organs at risk can be accurately located at the treatment angle enabling image guidance. Second, that the beam delivery system can accurately follow and adjust to gravity-induced motion in the patient anatomy and third that patients are able to tolerate horizontal rotation during treatment. This study primarily aims to address the first of these assumptions. Beam adaptation techniques shown described by Liu et al. [14] and a single institutional study (NCT02995603) that is currently underway to assess patient tolerance and acceptance have aimed to address the second and third assumptions, respectively.

Whilst deliberate movement of the patient is unique to the fixed-beam radiotherapy approach, patient motion (e.g. respiratory motion) is a universal radiotherapy problem. Many advances in modern radiotherapy have been geared towards tackling patient motion via both real-time imaging, to observe the motion, and real-time beam adaptation technologies such as MLC or couch tracking, to compensate for this motion. These motion management technologies would prove complementary to the fixed-beam, horizontal rotation approach. To the authors’ knowledge, this is the first study that will look at collecting CBCT images of patients undergoing horizontal rotation as part of a fixed-beam radiation therapy system.

An alternative approach to the horizontal rotation studied here would be a fixed-beam system that relies on upright rotation rather than horizontal rotation [21]. A seated or standing patient rotation system would mostly avoid gravity-induced motion and allow cone beam CTs to be reconstructed without motion blur. However, upright systems are not consistent with current radiotherapy workflows and equipment, which would require bespoke solutions for imaging and treatment planning.

One limitation of this study is that the prototype Nano X system does not allow complete translation of the couch under the treatment head and into the CBCT imaging field of view. Furthermore, the patient immobilisation on this couch cannot support patients in a feet-first orientation. As such the anatomical sites able to be imaged are limited to the head and neck, shoulders and upper torso due to the constraints of the current prototype system. This limitation is reflected in the limited selection criteria for this study. In addition, treatment sites such as the abdomen and pelvis, which are likely to exhibit more complex motion and deformation, are not able to be assessed. There is a risk that while motion compensation methods implemented for the reconstruction of patient Nano X CBCTs in this study might prove successful for the sites assessed, they may not be generally applicable to all anatomical sites.

## Data Availability

After study completion, de-identified (de-coded, non-identifiable) data may be shared with researchers for further scientific research separate from this study. Information about data sharing will be provided to study participants in the participant information sheet.

## Abbreviations

CBCT: Cone beam computed tomography
CT: Computed tomography
IG: Image guidance
MLC: Multi-leaf collimator
PRS: Patient Rotation System
